# Paradoxical suboptimal vitamin D levels in a Mediterranean area: a population-based study

**DOI:** 10.1038/s41598-022-23416-1

**Published:** 2022-11-16

**Authors:** D. A. Díaz-Rizzolo, B. Kostov, R. Gomis, A. Sisó-Almirall

**Affiliations:** 1 Health Science Faculty, Universitat Oberta de Catalunya (UOC), Barcelona, Spain; 2 Primary Healthcare Transversal Research Group, Institut d’Investigacions Biomèdiques August Pi i Sunyer (IDIBAPS), Barcelona, Spain; 3 Primary Care Centre Les Corts, Consorci d’Atenció Primària de Salut Barcelona Esquerra (CAPSBE), Barcelona, Spain; 4 Department of Statistics and Operations Research, Universitat Politècnica de Catalunya (UPC), Barcelona, Spain; 5 Universitat de Barcelona, Barcelona, Spain; 6 Diabetes and Obesity Research Laboratory, Institut d’Investigacions Biomèdiques August Pi i Sunyer (IDIBAPS) – Hospital Clinic of Barcelona, Barcelona, Spain; 7 Centro de Investigación Biomédica en Red de Diabetes y Enfermedades Metabólicas Asociadas (CIBERDEM), Madrid, Spain; 8 Department of Endocrinology and Nutrition, Hospital Clinic of Barcelona, Barcelona, Spain

**Keywords:** Endocrine system and metabolic diseases, Biomarkers, Endocrinology, Risk factors

## Abstract

Policies in sunny countries, such as those in the Mediterranean area, do not promote vitamin D supplementation despite some studies might suggest the high prevalence of sub-optimal levels. The objective was to determine the vitamin D levels by 25-hydroxyvitamin D (25(OH)D) of a Mediterranean population and their characteristics. This population-based study included a database of public health system from all individuals living in Catalonia > 18 years who had some measure of 25(OH)D between January 2018 and April 2021. More than half million people were classified based on 25(OH)D measurements to study their characteristics. Three vitamin D categories were created: < 20 ng/ml deficiency, 20–30 ng/ml insufficiency and > 30 ng/ml optimal. Less than 10% of the population residing in Catalonia had recent 25(OH)D determinations and the majority of determinations were in ≥ 45 years and in women. Around 80% of young people with determination had sub-optimal levels but the prevalence of vitamin D supplementation prescription increased with age which was associated with better values of 25(OH)D. In a Mediterranean area 25(OH)D determinations were low despite the high prevalence of suboptimal levels in the population with recent determination. In addition, the measurements were especially concentrated in people ≥ 45 years of age and in women who were, in addition, the groups to whom the most vitamin D supplementation was prescribed. On the contrary, young people presented few determinations of 25(OH)D and, although majority of them showed sub-optimal levels, vitamin D supplementation was not prescribed in most cases.

## Introduction

Over 80% of the world’s population has levels of circulating vitamin D, detected as 25-hydroxyvitamin D (25(OH)D), below desired^1^ and more than 40% of the population has deficiency in Europe^2^. There are vitamin D-rich foods, but the main natural way of obtaining this vitamin for humans is from endogenous production by solar exposure. For this reason, it makes sense to think in that countries in the Mediterranean area where access to solar exposure is easier than in the Nordic countries, vitamin D deficiency will be lower. However, the largest deficit prevalence is in the countries of southern Europe and the eastern Mediterranean^3,4^ probably due to the use of solar protectors and low consumption of vitamin D-rich food along with the absence of food fortification^5^. Historically, the highest risk groups of deficiency have been the elderly, with the highest risk of malnutrition, and the institutionalized people, with low solar exposure^5^. In Spain, the use of systemic vitamin D supplementation in the general population is not recommended^6^ and, specifically in Catalonia, a coastal region of Spain, the routine determination of 25(OH)D in the general population without symptoms is not recommended^7^.

Therefore, we collected information available from public health about all the people in Catalonia region who have recently 25(OH)D determination and used the data regarding gender, age and supplementation to examine the characteristics in relation to vitamin D in a Mediterranean area.

## Methods

This population-based study was approved by the ethics committee of Open University of Catalonia, Spain, and followed the Strengthening the Reporting of Observational Studies in Epidemiology (STROBE) reporting guideline.

The database is obtained from the PADRIS program (Public Data Analysis for Health Research and Innovation Program) which makes health data available to the scientific community through access to the reuse and crossover of health data generated by the integral health system of public use of Catalonia. The inclusion criteria for the data obtained were as follows: all individuals living in Catalonia > 18 years who had some measure of 25(OH)D between 1 January 2018 and 1 April 2021. The diagnostic vitamin D status classification was based on the Endocrine Society Clinical Practice Guideline^8^ following the 25(OH)D measures classified follows: < 20 ng/ml deficiency, 20–30 ng/ml insufficiency and > 30 ng/ml optimal. Regarding the statistical analysis, categorical variables are presented as absolute frequencies and percentages, and continuous variables are presented as the mean and standard deviation (SD). Participants were clustered according to age. Seventy-nine clusters with more than 1000 observations (from 19 to 97 years) were used to study the influence of age on 25(OH)D measurements. The statistical analysis was performed using R version 3.6.1 for Windows.

### Ethics approval

This was an observational study but for population data analysis, approval was obtained from the ethics committee of the Open University of Catalonia, Spain.

## Results

As shown in Table 1, a total of 519,118 people were included in the study. Less than 15% of 25(OH)D determinations were performed in people under 45 years of age (4% in the 18–29 yo and 11% in the 30–44 yo age range). The characteristics of the population where 25(OH)D is most determined and supplemented are ≥ 45 years. In addition, 76% of 25(OH)D measurements were determined in women.

**Table 1:** Population characteristics and distribution by vitamin D levels and supplementation.

Variables	All (n = 519,118)	Vitamin D supplementation	
		No (n = 262,684)	Yes (n = 256,434)
**Age, years**	64 ± 18	60 ± 18	69 ± 16
18–29	19,822 (4)	15,646 (6)	4,176 (2)
30–44	55,921 (11)	39,876 (15)	16,045 (6)
45–64	167,462 (32)	94,685 (36)	72,777 (28)
65–75	121,928 (23)	54,667 (21)	67,261 (26)
> 75	153,985 (30)	57,810 (22)	96,175 (38)
**Sex**			
Female	394,826 (76)	187,229 (71)	207,597 (81)
Male	124,292 (24)	75,455 (29)	48,837 (19)
**Vitamin D, ng/mL**	24 ± 13	22 ± 11	25 ± 14
Insufficiency	222,750 (43)	116,257 (44)	106,493 (41)
Deficiency	168,317 (32)	97,595 (37)	70,722 (28)
Normovitamin	128,051 (25)	48,832 (19)	79,219 (31)

Categorical variables are presented as absolute frequencies (percentages) and continuous variables as the mean ± standard deviation.

Suboptimal vitamin D levels, including all people with ≤ 30 ng/ml, were detected in 75% (391,067 pax) of the population and only 45% of them (177,215 pax) were treated with vitamin D supplements.

**Figure 1:**
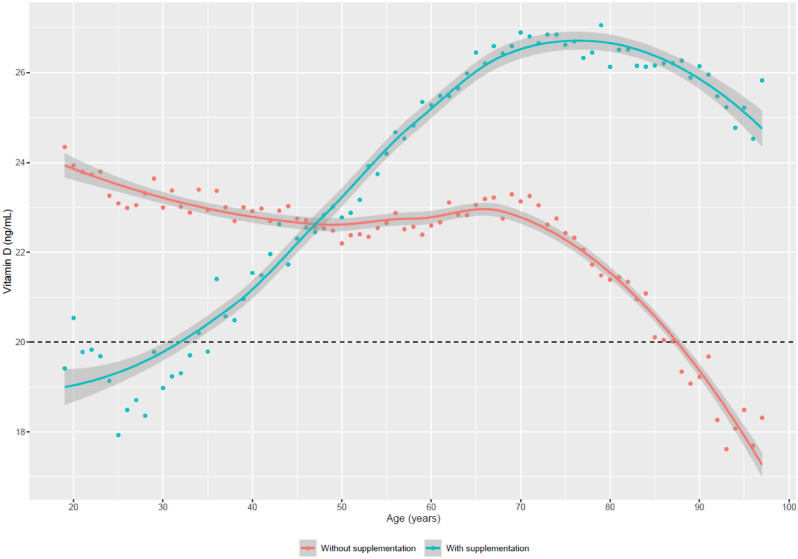
Vitamin D levels by age differentiated according to whether there is prescribed supplementation. The red and blue dots correspond to the averages of vitamin D in each age group without and with supplementation correspondingly. Smooth curves in red and blue color (with 95% confidence intervals) represent the association between age and vitamin D.

Figure 1 shows that the average 25(OH)D at each age decreases naturally without associated supplementation, but the opposite trend is observed in the supplemented group. The age groups most affected by vitamin D deficiency are the extreme ones, with supplementation being the youngest and without supplementation being the oldest (Fig. 1). Specifically, the distribution of deficiency increases with age when there is no supplementation and the opposite occurs when there is supplementation (Table 2). Although it can be seen how decisive vitamin D supplementation is, among the population without supplementation, only between 17 and 22% have optimal 25(OH)D values according to the age categories.

**Table 2:** Population categorization according to vitamin D level group and presence of supplementation.

Variables	Without vitamin D supplementation (n = 262,684)				With vitamin D supplementation (n = 256,434)			
	Vitamin D, ng/mL	Insufficiency (n = 116,257)	Deficiency (n = 97,595)	Optimal (n = 48,832)	Vitamin D, ng/mL	Insufficiency (n = 106,493)	Deficiency (n = 70,722)	Optimal (n = 79,219)
**Age, years**		62 ± 19	58 ± 17	59 ± 18		67 ± 17	69 ± 15	71 ± 14
18–29	24 ± 10	5,854 (37)	6,392 (41)	3,400 (22)	19 ± 12	2,723 (65)	827 (20)	626 (15)
30–44	23 ± 10	15,640 (39)	16,498 (41)	7,738 (20)	21 ± 13	9,191 (57)	3,842 (24)	3,012 (19)
45–64	23 ± 10	39,655 (42)	37,802 (40)	17,228 (18)	24 ± 14	32,222 (44)	20,497 (28)	20,058 (28)
65–75	23 ± 11	22,988 (42)	20,982 (38)	10,697 (20)	27 ± 14	24,104 (36)	20,250 (30)	22,907 (34)
> 75	21 ± 13	32,120 (55)	15,921 (27)	9,769 (17)	26 ± 16	38,253 (39)	25,306 (27)	32,616 (34)
**Sex**								
Female	23 ± 11	79,676 (43)	71,066 (38)	36,487 (19)	26 ± 15	83,906 (40)	58,168 (28)	65,523 (32)
Male	21 ± 10	36,581 (49)	26,529 (35)	12,345 (16)	24 ± 14	22,587 (46)	12,554 (26)	13,696 (28)

Categorical variables are presented as absolute frequencies (percentages) and continuous variables as the mean ± standard deviation.

## Discussion

Catalonia has an adult population of > 6 million inhabitants^9^ and 25(OH)D levels have only recently been studied in approximately half a million which supposes a detection of < 10% of the total adult population. This low rate of the 25(OH)D test makes sense according to the regional^7^ and state^6^ recommendations, which only recommend measurements in the population at risk defined as hospitalized people, institutionalized elderly individuals, people with prolonged immobilization, those with neoplastic diseases, those with other skin diseases that should not be exposed to the sun or those with gastrointestinal malabsorption and pregnant women.

Our results show that measurements of 25(OH)D as well as vitamin D supplementation are not only focused on the population ≥ 45 years of age, but also especially on women, which does not correspond to the risk groups for deficiency described by the European Society of Endocrinology, who also recommends more population measurements to know the status of vitamin D in Europe^5^.

While the young people who were measured presented high rates of deficiency, the prescription of vitamin D supplementation was very low. This is consistent with the conclusion, with evidence rating B, that treating asymptomatic individuals with identified deficiency has not been shown to improve health^10^. Nevertheless, there is a reverse J-shaped association between 25(OH)D and all-cause mortality^11^ and there could be specific symptoms or nonskeletal disease states for which vitamin D measurement and supplementation improve health. Specifically, the need to create more defined randomized clinical trials not only with with larger populations^12^ but also with more specific designed and better analyzed, has become evident^13^ as has already been shown for 25(OH)D and vitamin D supplementattion^14^.

## Limitations

This study was designed according to a ‘Big Data’ approach and has some limitations associated with its retrospective design, inclusion bias and codification errors. Despite these limitations, 25(OH)D measurements of more than half million people allow robust conclusions to be reached on the relationship between vitamin D measurements, age, sex and supplementation.

The inclusion of the study determined by the existence of a recent measurement of 25(OH)D defines our population with an inclusion bias since, especially among young people, they may be people with symptoms of vitamin D deficiency. Therefore, in this case we cannot think of the average 25(OH)D values by age and sex as representative values of the population.

## Conclusion

Our study found that the proportion of inhabitants in a Mediterranean area whose vitamin D was determined to have suboptimal level was > 75%. In fact, the population ratio with recent 25(OH)D determinations was low and the characteristics of people with recent determination did not meet national recommendations of risk group control. In addition, the determination in the younger groups was especially scarce and, despite presenting suboptimal levels of 25(OH)D, this was not associated with the prescription of supplements.

These findings suggest that, despite residing in a sunny area, increasing 25(OH)D determinations could be beneficial for the population, especially if the diagnosis of suboptimal levels is related to the prescription of vitamin D supplements.

## Data Availability

The data that support the findings of this study are available from the PADRIS program but restrictions apply to the availability of these data, which were used under license for the current study and thus are not publicly available. Data are, however, available from the corresponding author upon reasonable request and with permission of PADRIS program [https://aquas.gencat.cat/ca/ambits/analitica-dades/padris/].

## References

[1] Hilger J, Friedel A, Herr R, Rausch T, Roos F, Wahl DA (2014). A systematic review of Vitamin D status in populations worldwide. Br. J. Nutr..

[2] Cashman KD, Dowling KG, Škrabáková Z, Gonzalez-Gross M, Valtueña J, De Henauw S (2016). Vitamin D deficiency in Europe: Pandemic?. Am. J. Clin. Nutr..

[3] Elrayah EE, Rogers L, Doggui R, Al-Jawaldeh A (2020). Vitamin D insufficiency and deficiency in the eastern Mediterranean region (EMR)-misconceptions in public health practice: A scoping review 2019–2020. J. Nutr. Sci. Vitaminol..

[4] Grant WB (2019). Vitamin D and health in the Mediterranean countries. Hormones.

[5] Lips P, Cashman KD, Lamberg-Allardt C, Bischoff-Ferrari HA, Obermayer-Pietsch B, Bianchi ML (2019). Current vitamin D status in European and Middle East countries and strategies to prevent vitamin D deficiency: A position statement of the European Calcified Tissue Society. Eur. J. Endocrinol..

[6] Sociedad Española de Endocrinología y Nutrición (SEEN). La insuficiencia de vitamina D es una epidemia mundial, que afecta a más de la mitad de la población. Available: https://www.seen.es/ModulGEX/workspace/publico/modulos/web/docs/apartados/783/240320_113242_2463317370.pdf (2018).

[7] Generalitat de Catalunya (GENCAT). Niveles séricos de vitamina D. 2018 available: http://essencialsalut.gencat.cat/es/detalls/Article/vitaminad_nivells_serics

[8] Holick, M. F., Binkley, N. C., Bischoff-Ferrari, H. A., Gordon, C. M., Hanley, D. A., Heaney, R. P., Murad, M. H. & Weaver, C.M. Evaluation, treatment, and prevention of vitamin D deficiency: An Endocrine Society Clinical Practice Guideline. *J. Clin. Endocrinol. Metab.* **96**(7), 1911–1930. 10.1210/jc.2011-0385 (2011). Erratum in: J. Clin. Endocrinol. Metab. **96**(12), 3908. PMID: 21646368. 10.1210/jc.2011-0385 21646368

[9] Instituto de Estadística de Cataluña (IDESCAT). www.idescat.cat visited 26th January 2022 with last update 20th December 2021.

[10] LeFevre ML, LeFevre NM (2018). Vitamin D screening and supplementation in community-dwelling adults: Common questions and answers. Am. Fam. Physician.

[11] Sempos CT, Durazo-Arvizu RA, Dawson-Hughes B, Yetley EA, Looker AC, Schleicher RL, Cao G, Burt V, Kramer H, Bailey RL, Dwyer JT, Zhang X, Gahche J, Coates PM, Picciano MF (2013). Is there a reverse J-shaped association between 25-hydroxyvitamin D and all-cause mortality? Results from the U.S. nationally representative NHANES. J. Clin. Endocrinol. Metab..

[12] Pilz S, Trummer C, Theiler-Schwetz V, Grübler MR, Verheyen ND, Odler B, Karras SN, Zittermann A, März W (2022). Critical appraisal of large vitamin D randomized controlled trials. Nutrients.

[13] Heaney RP (2014). Guidelines for optimizing design and analysis of clinical studies of nutrient effects. Nutr. Rev..

[14] Dawson-Hughes B, Staten MA, Knowler WC, Nelson J, Vickery EM, LeBlanc ES, Neff LM, Park J, Pittas AG, D2d Research Group (2020). Intratrial exposure to vitamin d and new-onset diabetes among adults with prediabetes: A secondary analysis from the vitamin D and type 2 diabetes (D2d) study. Diabetes Care.

